# Finding their place – general practitioners' experiences with palliative care—a Norwegian qualitative study

**DOI:** 10.1186/s12904-022-01015-1

**Published:** 2022-07-12

**Authors:** Anne Fasting, Irene Hetlevik, Bente Prytz Mjølstad

**Affiliations:** 1 General Practice Research Unit, Department of Public Health and Nursing, NTNU, Norwegian University of Science and Technology, PO Box 8905 MTFS, N-7491 Trondheim, Norway; 2 Unit for Palliative Care and Chemotherapy Treatment, Cancer Department, More Og Romsdal Hospital Trust, Kristiansund Hospital, Kristiansund, Norway; 3 Saksvik legekontor, Saxe Viks veg 4, N-7562 Hundhammeren, Norway

**Keywords:** Palliative care, Primary care, Palliative medicine, General practice, Advance care planning, End-of-life care, Transitions of care, Norway

## Abstract

**Background:**

Modern palliative care focuses on enabling patients to spend their remaining time at home, and dying comfortably at home, for those patients who want it. Compared to many European countries, few die at home in Norway. General practitioners’ (GPs’) involvement in palliative care may increase patients’ time at home and achievements of home death. Norwegian GPs are perceived as missing in this work. The aim of this study is to explore GPs’ experiences in palliative care regarding their involvement in this work, how they define their role, and what they think they realistically can contribute towards palliative patients.

**Methods:**

We performed focus group interviews with GPs, following a semi-structured interview guide. We included four focus groups with a total of 25 GPs. Interviews were recorded and transcribed verbatim. We performed qualitative analysis on these interviews, inspired by interpretative phenomenological analysis.

**Results:**

Strengths of the GP in the provision of palliative care consisted of characteristics of general practice and skills they relied on, such as general medical knowledge, being coordinator of care, and having a personal and longitudinal knowledge of the patient and a family perspective. They generally had positive attitudes but differing views about their formal role, which was described along three positions towards palliative care: the highly involved, the weakly involved, and the uninvolved GP.

**Conclusion:**

GPs have evident strengths that could be important in the provision of palliative care. They rely on general medical knowledge and need specialist support. They had no consensus about their role in palliative care. Multiple factors interact in complex ways to determine how the GPs perceive their role and how involved they are in palliative care. GPs may possess skills and knowledge complementary to the specialized skills of palliative care team physicians. Specialized teams with extensive outreach activities should be aware of the potential they have for both enabling and deskilling GPs.

**Supplementary Information:**

The online version contains supplementary material available at 10.1186/s12904-022-01015-1.

## Background

### Family medicine and palliative care

Palliative care in Europe is based on a comprehensive care philosophy comprising a holistic approach in a multi-disciplinary and multi-professional setting [1]. The patients can have symptoms that require simple or complex medical treatment and nursing care but may also suffer on a spiritual and existential level. Cicely Saunders, who first introduced this model of thinking, described this as “total pain”. The contemporary multi-disciplinary approach of palliative care focuses on the physical, mental, social, and spiritual aspects of suffering, including the family perspective [1, 2]. These principles are applicable to all patients with life-limiting illness and may also be relevant early in the disease [1, 3].

The palliative culture’s approach to the patient resembles the approach of the general practitioner (GP) in many respects [4]. Family medicine and general practice are, by their very nature, person-centred and based on a bio-psycho-social understanding of illness [5–7]. At the same time, GPs are generalists, and they relate to the entire life course from birth to death, including palliative care at end-of-life [8].

These two approaches differ somewhat from other medical fields and the specialities in the hospitals, where specialization may lead to fragmentation of treatment and care [9].

### The GP’s role in palliative care

As most patients prefer to spend as much time as possible and die in their own home, the amount of home deaths is considered an important quality indicator for palliative care [3, 10–12]. In Norway, less than 15 percent of deaths take place in the home [13], and this is a low rate compared to other European countries [14]. Previous research suggests that GPs’ participation in this work could contribute to increase this rate [3, 15–20].

In Norway, the GP serves as the point of first contact and coordinator for healthcare, and access to specialist services requires a referral from a GP. By means of a listing system, all citizens are entitled to have a regular general practitioner (RGP) [21]. An RGP must be either a specialist in general practice, or in training for the speciality [21, 22]. There is a Continuing Medical Education program (CME), where groups of GPs meet regularly to maintain their competence. Beyond basic education, there is no mandatory curriculum in palliative care to practice as an RGP in Norway, or to become a specialist in general practice [22].

Palliative care in Norway is reinforced by specialized, multidisciplinary palliative care teams (PCTs). They are mainly hospital-based, work ambulatory towards primary care and has an advisor role [3]. Thus, the GPs are formally responsible for the medical care and the PCTs should not normally provide total care for the palliative patients residing at home [3].

GPs are described as missing in the palliative care trajectories, and difficult to integrate into the palliative care networks in primary care [23, 24]. We previously conducted a questionnaire study of GPs in Norway, finding that most GPs had few palliative patients at a time and that being involved in home death was a rare event, limiting their possibility of learning and maintaining complex skills and knowledge of palliative care [25]. Yet, about half of the RGPs saw themselves as central in providing palliative care in the primary care setting, challenging the prevailing views of the Norwegian GPs’ as “missing” or uninterested in palliative care [23]. These findings suggest variation in GPs’ involvement in palliative care that remains unexplained at this point.

The aim of this study is to explore GPs’ experiences in palliative care regarding:

- Their involvement in this work.
- How they define their role.
- What they think they realistically can contribute towards these patients.

## Methods

### Design

We aimed to explore experiences and perceptions, and thus a qualitative approach was chosen. Focus group interviews are deemed a quick and convenient way to gather data from several people and have the advantage that group interaction often stimulates good discussions [26]. We expected the group dynamics to further illuminate the variable attitudes and experiences we aimed to investigate. A semi-structured interview guide was designed to ensure that the same topics were explored in all interviews. The data for this paper are drawn from the first part of the interview guide exploring experiences with and role in palliative care (Additional file 1), whereas the second part of the interview provides the basis for another paper not yet published, focusing on barriers towards guideline implementation. We wanted to explore the GPs’ experiences of working with palliative care, followed by a discussion about their role in palliative care. The questions were open-ended and the order flexible. Related topics raised spontaneously were explored, and the participants could revisit previous topics if needed.

### Participants, setting, and data collection

We approached established CME groups of RGPs and one tutorial group of RGP trainees. The groups were purposively recruited, aiming to include RGPs from both urban and rural settings, with different lengths of experience, and with variation in gender and age. The RGPs were affiliated with four different hospitals, and thus different PCTs. Groups were located by identifying the group administrators and were subsequently approached by AF personally and included if all the members agreed to participate. In this process, one group declined due to lack of time. Each individual participant gave written consent. For reasons of convenience, we invited groups from two counties in Mid-Norway to participate.

From February to June 2018 four groups with a total of 25 participants were interviewed once, at a location of their choice. The median age was 42. The mean patient list length was 1,032, and the mean amount of experience in general practice was 10.5 years. Groups 1 – 3 were CME groups. The fourth group was the tutorial group where the tutor was a specialist in general practice. The participants in this group were younger, with a median age of 32, excluding the tutor.

Details of the demographic and professional data are given in Table 1.

**Table 1:** Characteristics of the participating GPs (1-25) in groups (1-4)

**Group**	**GP number**	**Gender**	**Age**^b^	**Practice**	**List length**^b^	**Setting**	**Specialist**	**Years as GP**
**1**	1	M	40–45	Group	1400	urban	Yes	10
	2	M	40–45	Group	1450	urban	Yes	15
	3	M	40–45	Group	1200	urban	Yes	13
	4	F	45–50	Group	1100	urban	Yes	9
**2**	5	M	60–65	Group	1000	urban	Yes	29
	6	F	45–50	Group	1000	urban	Yes	15
	7	M	45–50	Group	1000	urban	No	10
	8	F	45–50	Group	600	urban	No	7
	9	M	65–70	Solo	700	urban	Yes	39
**3**	10	M	55–60	Group	1050	rural	Yes	8
	11	F	35–40	Group	1000	rural	Yes	6
	12	M	35–40	Group	1000	rural	No	6
	13	F	40–45	Group	1200	rural	Yes	11
	14	F	30–35	Group	550	rural	No	4.5
	15	M	60–65	Group	1000	rural	Yes	30
	16	M	40–45	Group	1000	rural	Yes	12
**4**	17	M	30–35	Group	900	rural	No	4.5
	18	F	30–35	Solo	1000	rural	No	2
	19	M	30–35	Group	1000	urban	No	1
	20	M	35–40	Group	1100	rural	No	3
	21	M^a^	45–50	Group	1100	rural	Yes	15
	22	F	30–35	Group	1350	urban	No	2
	23	F	25–30	Group	1050	rural	No	3
	24	M	30–35	Group	1300	rural	No	3.5
	25	F	30–35	Group	750	rural	No	3.5

*M* Male, *F* Female

^a^Group tutor

^b^Age given in intervals and list lengths rounded to ensure anonymity of participants

The interviews were moderated, recorded on audio tapes, and transcribed verbatim by AF. An experienced qualitative researcher participated as support and observer of the interviews, while also providing extensive field notes of the sparce non-verbal expressions of interest for the discourse. For each interview, the content was compared with the previous interviews and field notes in search of new topics. In the fourth interview new relevant topics did not evolve and the data was deemed as saturated, holding sufficient information power to illuminate our research questions [26, 27]. The interviews progressed in a calm manner, differences of opinions were welcomed with interest, and the participants politely gave room for each other in the discussion, with no overt negative emotions. The groups raised questions resulting in the discussion of topics not covered by the interview guide, such as doctors’ delay and ethical considerations.

### Analysis

We performed a qualitative analysis inspired by interpretative phenomenological analysis (IPA) described by Smith [28–30]. The transcribed interviews were re-read several times by AF and BPM separately for an overall impression. We then worked through the transcripts, noting interesting topics and thoughts. The use of language was reflected on. Based on this, codes and emergent themes were identified for each interview and connections across themes explored. When all the interviews were thus analysed, AF and BPM discussed patterns across the interviews, looking for superordinate, shared themes. We then applied the same approach, following each GP’s voice through the interviews, with the intention to capture the particular (idiographic) accounts of individuals [28, 29, 31]. Author IH read and analysed the interviews independently, cross-checking whether identified themes corresponded with the overall impression from the interviews.

To enhance credibility and confirmability of the study, preliminary results were presented and discussed in different forums of peer researchers, GPs, and palliative care physicians.

The data was initially handled in the NVivo software and then transferred to Microsoft Word documents for the completion of the analysis.

## Results

The material yielded rich descriptions of what the GPs perceived as their strengths in providing palliative care. Positive attitudes prevailed in all the groups, but when it came to their formal role in palliative care, no consensus emerged, as the GPs took differing positions. Below we present the findings with some illustrative examples.

### Strengths of the GP in providing palliative care

The GPs highlighted characteristics of general practice that they believed were significant for their provision of palliative care, as well as relevant skills they relied on in this work.

In all the interviews, the GPs expressed confidence that they had *general medical knowledge*, sufficient to provide basic palliative care, as described by this GP:

GP 1: “But pain, nausea, constipation, ordinary palliative symptom relief, are problems I think many of us can deal with.”

The GPs thought that providing *continuity of care* could be important for their palliative patients. They described following their patients over several years, through various medical diagnoses and events. Having *personal knowledge* of both the patients and their families was regarded as unique for the GP and included knowledge about the patient as a person (personal traits, behavioral responses, hobbies of interest) as well as important life events. This relationship was also seen as important for the feeling of *safety for the patient*. Doctor 9 put it like this:

GP 9: “It can be quite reassuring to have a doctor who knows the patient. In many cases, that doctor has treated the patient for many years, and may be more than just some random doctor to them. They see you as a real person. At least I can say that many of my patients have been my patients since I started practising. That means we know each other well.”

Also, being *able to console* the patient and family when entering the palliative trajectory was highlighted as an important, yet challenging, task. One GP put it like this:

GP 21: “Usually when we console people in private like that, we tell them that everything is going to be okay. Don’t worry, it will be fine. But under these circumstances, you can’t say that, so you have to think of something else to say to them, that is, you have to come up with a different story. Then you have to be able to say something like ‘We’re going to do everything we can going forward.’ That's what we'll do. You have to give them something in this situation, right?”

Several of the GPs stated that they were *able to deal with the existential needs* of the patients and relatives. The GPs disussed how they saw it as an important, yet challenging, task to help the patients to come to terms with a serious diagnosis and a poor prognosis. As one GP described:

GP 13: “It can be a bit challenging to get the patient to concentrate on the right things early on, rather than putting things off. I don’t want to be negative about the prognosis, but I know it’s bad, and that things can take a turn for the worse quickly, so it’s important to think through these things and to decide what is important. I find that challenging.”

Being the GP of family members also positioned them to *provide psychosocial care* for the whole family, not necessarily thinking of it as providing palliative care, but as part of their everyday work. Many reported to be RGP for several family members like spouses, parents, and children:

GP 22: “Yes, I think it was a little easier, maybe because I was, and still am, the whole family’s RGP. Because of that, I saw them more frequently, like when the children were sick, that is, her grandchildren. And it was only natural that I discussed the mother then.”

The GP’s position as *coordinator of care* was seen as valuable for palliative patients and particularly important in longer trajectories, elderly patients, and non-cancer diagnoses. The GPs stressed the importance of receiving realistic prognostic information to be able to recognize patients as palliative. RGPs receive discharge summaries from all the different specialists in hospitals and need this information, as highlighted by this GP:

GP 24: “But when they’re just sitting there at the hospital, in front of the hospital doctor, sort of nodding their heads and trying to look like they understand, well, then maybe they can’t even manage to react because they’re in shock. I’ve experienced several times that they have come to me afterwards and said that they want to come to me every time to review the medical records in question, because I tell them what the records mean, to give them a better understanding.”

### How GPs perceive their role in palliative care

#### Having generally positive attitudes

When it came to attitudes towards palliative care as a field, the GPs were generally positive. Although most expressed some ambivalence related to the demands, it was seen as *rewarding work* and something from which the GP would benefit both personally and professionally:

GP 10: “Of course, it's demanding, but it's also challenging in both medical and human terms, and it's interesting. You get really close to the patient, and sometimes even to the relatives. Sometimes I almost feel like I'm part of the family, especially towards the end, when there is fairly close follow up. Yes, it is a special situation, but I often find it a rewarding part of the RGP-patient relationship.”

They particularly highlighted the importance of being able to end a long-term doctor-patient relationship in a good way, fulfilling a need for closure. Although participation in planned home death varied greatly, this was highlighted as an ideal by GPs in all the interviews, as in this exchange from interview 2:

GP 6: “I was on an emergency, out of hours house call yesterday, to see a patient who was allowed to die at home. There was a tremendous sense of calmness and serenity under the circumstances.” GP 8: “Yes, there is great dignity when they can be allowed to stay at home, as long as the relatives can handle it. Being in safe, familiar surroundings is really wonderful, in my opinion.”

As the work was seen as valuable, some of the GPs expressed a sense of loss when they perceived that care of the patient “disappeared” into the hands of others. Also, there seemed to be a transition over the years where some GPs had lost some of their tasks to others:

GP 9: “But I also feel, like GP 5 said, that we have lost a little ground. Considering some of the other things we're required to do, I think maybe this would be rather more worthwhile than a great deal of the other [things we do]. It would be prudent for us to maintain our expertise in this, and I think it would also be worthwhile for many patients as well.”

#### Describing their role – three positions towards palliative care

Whereas attitudes were generally positive, views about their formal role in this work varied. Across the interviews, no consensus emerged concerning the GP’s role and how much they thought the GP should participate in palliative care. The different accounts followed three main patterns. We interpreted this as the GPs displaying different levels of involvement with palliative care. Although this involvement must be understood as ranging over a continuum, and not all the individual GPs’ accounts contained enough information to be thus classified, three illustrative positions towards palliative care emerged: the highly involved, the weakly involved, and the uninvolved GP. The three positions, with their key characteristics, are presented and illustrated below:

### The highly involved GP

GPs of this category were found in groups 3 and 4. They were represented by both older and younger GPs, specialists, and non-specialists, and both genders. Additionally, they all worked in rural environments.

The highly involved GPs described themselves as *the key worker* in palliative care in their community; they participated regularly in this work and would prioritize these patients. They thought of palliative care as a natural part of their job. They also described how they regularly participated in terminal care at home almost as a normal, everyday event:

GP 18: “Well, I’ve had a few patients over the years. There were two home deaths last week, I think.”

These GPs described themselves as being in charge and saw other trajectory participants as resources they could draw on. Cooperation was described according to predictable patterns, and the GPs were confident where to get help, both from hospital specialists and the community nursing service.

GP 16: “I think it would have been difficult to have a good death at home without an RGP involved, assuming the role of primary actor. You can use the palliative team as a resource, and the community nursing team can also be a valuable resource for implementation and observation, but in any case, the RGP is right in the thick of things, exactly where he or she has to be to make this work, in my opinion.”

These GPs described the presence of *clear clinical handover* processes from the hospital specialists, especially for cancer patients. The handover was typically signalled by cessation of curative treatment, as described by this GP:

GP 16: “It’s fairly common to have an attending oncologist who's been in charge of the patient throughout the course of the curative treatment. Then at some point, the oncology department decides that it’s time to discontinue the curative treatment and move on to palliative treatment. I experience this transition as being very clear.”

The highly involved GPs described how they proactively claimed a role or reclaimed the patient when entering the palliative phase. They also highlighted the importance of advance care plans (ACPs), and would make themselves available out of hours (OOH):

GP 15: “I've been involved in many palliative situations. I feel like the most beautiful deaths, the best for the patient and relatives alike, have been when people die at home. However, they have also been the best planned, most thoroughly organised deaths. Me being available on my mobile phone gives a sense of security to the patient, the home care team, and the relatives. However, I very rarely get such calls. I've never been rung up at night, and only a few times on evenings and weekends. When I have been contacted, it's been nice because things can be resolved quickly by phone.”

### The weakly involved GP

GPs of this category were found in interviews 1 and 4. They were of varying gender and age (although none were over 50 years of age), both specialists and non-specialists, and worked in urban or rural environments.

The weakly involved GPs expressed ambivalence about what their role in palliative care was and debated whether other participants might do a better job, thus questioning their own ability to provide total care. They spoke about the PCTs as in charge of the palliative cancer patients and expressed unsureness about who was in charge in the case of non-cancer diagnosis. They rarely participated in planned home death and described how other professionals took over care and how they lost track of the patients:

GP 2: “The cancer patients are quickly taken over by a palliative team at the hospitals that often do the emergency house-calls too.” GP 4: “Absolutely!”

They displayed variable involvement in palliative care, often associated with specific circumstances. Having *a prior close doctor-patient relationship* was given as a factor in increasing involvement. They could be *actively involved by other participants* in the trajectory as described by this GP:

GP 22: “I actually played quite an important role at those times. But the municipal oncology nurse was in charge, and she called me in when they had meetings. And I always visited the family as well.” Interviewer: “So, in other words, you were encouraged to play an active part in the process?” GP 22: “Yes. And it was actually very rewarding.”

These GPs described being more involved if *no clear hospital specialist was in charge*, e.g., when the patient had several illnesses and did not suffer from cancer. This would typically be older patients with longer, more unpredictive palliative trajectories. This GP described such a case where the patient was multimorbid:

GP 3: “I have a totally different story as well, about a time when I was left sitting with everything all on my own. But that wasn’t cancer. There was no hospital specialist, or whoever. Maybe I could have consulted the people in the stroke unit when she was there, but it was what it was.”

The weakly involved GPs described a *less clear clinical handover process* for palliative care patients than the highly involved GPs. This topic included the *division of labour* and *quality of the information* transferred from the specialists. An ad hoc negotiation in the service from case to case, with no clear system, was also described – typically, the GP would perform the tasks if no one else would, as expressed by this GP:

GP 3: “When time is at a premium, I find myself dodging or skipping things, if there are others who can handle them. I step up when I have to, though.”

The weakly involved GPs were less inclined to be proactive than the highly involved GPs. They were ambivalent and presented reasons to not to contact the patient, take charge or make themselves available:

GP 21: “I think it is very important, because it is about protecting ourselves a little and having some time off. We can of course work constantly. Like, work every day, seven days a week, and never take a day off. I think it's important that we can tune out occasionally and take some time off.”

### The uninvolved GP

GPs of this category were mainly found in interview 2. They were both older GPs and younger and could be specialists or non-specialists. They all worked in an urban environment.

The uninvolved GPs typically thought of palliative care as something GPs were little involved in and thought that these tasks belong with the palliative teams or other specialists. They described inconsistent involvement in palliative trajectories, and this mainly happened if the specialist in charge was not available – an exception from the “normal.” Contact with the patient was described as lost when the patient disappeared into the hands of the hospital specialists. They did, however, describe more involvement in patients with non-cancer diagnoses or longer palliative trajectories. They reported sporadic involvement in planned home death, if at all:

GP 9: “It was a pure coincidence because the palliative care team was away, and I was contacted to make a house call. So, I went to see him, and it was a nice experience for both of us. He died the following week. But if the team not been gone [on summer holiday], we wouldn’t have had that encounter.”

Cooperation seemed unpredictable to these GPs, and they didn’t seem to know the structure of the palliative care services well, as evident from this statement:

GP 5: “I can just say, from my perspective, that our role in this has diminished significantly over the years. This has happened as the municipal teams have evolved, and the hospital also has a group, doesn’t it?”.

They presented strong and compelling reasons not to be proactive. They pointed to the very nature of general practice, having no tradition for outreach activities, and pointed to the boundaries of their working hours. They discussed whether it was ethically appropriate to prioritise patients with palliative needs over other groups of vulnerable patients. They also found it problematic to invite themselves into the patient's home for a house call and then to charge them afterwards, as illustrated in the following quote:

GP 6: “We are, in point of fact, self-employed. It might sound silly, but it strikes me that I have a financial incentive for making house calls and I would like them to call. Or I could ring them up and ask if they would like me to come. But I’m not going to just show up, ring the bell and say: ‘Here I am.’ To be clear, this discussion is not just about palliative patients. There are no doubt many patients who might appreciate us reaching out to them.”

## Discussion

### Main findings

This study investigates GPs’ experiences in palliative care concerning their role and involvement, from their own point of view. Whereas GPs generally had positive attitudes, they also saw working with palliative care as demanding. The participating GPs pointed to various aspects of being a GP as their strengths in palliative work. They highlighted elements of the structure of general practice as important, including characteristics such as a longitudinal relationship with the patient, unique knowledge of both patient and family, and the GP as coordinator of care, representing continuity of care. They reported to have skills to provide basic symptom relief due to possessing general medical knowledge and the ability to provide psychosocial as well as existential care for their seriously ill patients, but they also relied on support from the specialized PCTs. The PCTs were seen as mainly serving cancer patients, whereas getting specialist support for multimorbid patients was more difficult. At the individual level, the GPs displayed different positions towards their role in palliative care, from the highly involved GP who feels central to the palliative care process, through the weakly involved GP, to the GP who is uninvolved in palliative care. There was a rural – urban difference, with rural GPs being more involved in palliative care than their urban colleagues.

### Strengths and limitations

Steps were taken to ensure trustworthiness of our results [32]. Consistency of results was ensured by author IH reading the interviews independently. Discussion of preliminary results with peers adds to the credibility of our results. Interpretative phenomenological analysis rests on a firm theoretical framework, a well described method, and a focus on extensive reflexivity, adding to the dependability and confirmability of findings [30]. To increase transferability of results, we have provided rich descriptions of the research setting, and our results are accompanied by direct quotes [32].

AF previously worked as an RGP and is currently working as palliative care consultant and BPM and IH are both experienced RGPs. Our experience gave us valuable insights and access to the field of interest. AF did not have previous knowledge of the groups beyond being acquainted with some of the participants from other professional settings. The moderator's role and how this could affect the group discussion became the subject of reflection in the analysis process supported by field notes, as described above.

Recruitment of GPs for research purposes is known to be difficult, and willingness to participate may be influenced by the GPs’ interest in the subject studied [33]. Approaching established groups of GPs not only eased the inclusion, but recruitment at the group level also allowed for the inclusion of GPs without special interest in the topic. Restricting recruitment to one geographic region raises issues of representability; on the other hand, it allowed for purposeful and strategic sampling within this region. In Norway, the structure of the health regions is similar, and our sample of GPs does not differ significantly from other Norwegian GPs in terms of age, gender, and experience level, and we believe our results could be representative for many Norwegian counties.

The participants were peers and represented a fairly homogenous group of professionals. As these were pre-existing groups, the familiarity between the participants allowed them to reflect openly and express themselves freely. In our view, this reduces concerns about group dynamics challenging the validity of analysing individual accounts within the material. Although the individual voices in a focus group must be interpreted in the light of the group context, the application of an IPA-approach to focus groups has been successfully conducted by several authors, and we believe it supported the exploration of individual (ideographic) aspects in our material [28, 29, 31].

Whereas focus groups stimulate discussion, it also opens for biases of self-presentation and social desirability [34]. For instance, expressions of strong positive attitudes towards palliative care could be exaggerated within the group, thus hindering views that conflicted with this. We did not, however, uncover any overt signs of this during the analysis [34]. The existing social ties of the group could also aggravate evaluation apprehension or normative influences [35]. This was particularly relevant for the fourth group, as the senior tutor took part in the group discussion, potentially taking a lead. This potential was considered before the interview and steps were taken to encourage all participants to take part in the discussion.

### Findings in the light of current knowledge

The Norwegian healthcare structure has similarities with many countries in Europe, and our finding may be of particular relevance to countries with a similar listing system for GPs [21, 36–38]. The planning of palliative care in rural areas is recognized as a challenge in several countries, to which our findings about rural GPs may be relevant [39, 40].

Issues of GPs’ participation in palliative care have been addressed in previous studies, and barriers such as resource concerns, access to palliative care expertise, or lack of formal training and knowledge have been identified [41]. The importance of GPs’ participation in the palliative care trajectory, particularly when it comes to increasing time and planned home deaths, has been demonstrated by various authors both prior to [15–17, 19, 20] and contemporary with our study [42, 43]. Our findings add to this evidence by demonstrating that GPs have abilities and are aware of important strengths they could contribute to palliative care processes.

A key feature highlighted by the GPs in our material was the continuity of care provided by the GP, which is in line with previous findings [44, 45]. In a Norwegian study in 2020, likeliness of a home death increased with the number of home visits from the GP, whereas having to leave the home for GP consultations, OOH-services, or hospital admission was associated with a reduced likeliness of a home death [43]. A recent Danish study showed increased home death rates independently of the number of contacts with the GP in a clinic that adopted an active and structured approach to palliative patients [42], indicating that there is a link between the mere involvement of GPs in the palliative trajectories and the likeliness of achieving home death. A long-standing GP-patient relationship is known to reduce the use of OOH-service and hospital admissions in the general population [46]. Furthermore, it is known that continuity of care in primary care is important when organizing palliative care [45, 47], and according to a systematic review from 2021, the lack of continuity of care is associated with end-of-life hospital admissions OOH [48]. A recent Norwegian study found that GPs find it hard to avoid OOH hospital admissions if they have not been involved in the care of the patients [49]. In correspondence with this, our GPs could be right in thinking that the continuity they provide may be a particularly important contribution towards the palliative patients.

Our material suggests that the general medical knowledge that GPs possess could enable them to provide symptom control for many patients at the end-of-life. Most dying patients do not need specialized palliative care to achieve symptom control [50, 51]. Previous findings indicate that GPs are familiar with the treatment of symptoms that are frequent in palliative care and have skills to provide basic palliative care, whereas they do not seem to have the same awareness of the treatment of more uncommon symptoms, and bereaved relatives perceive patient outcome as poorer compared with other care settings [52, 53]. Also, GPs’ skills and knowledge in palliative care has been shown to vary [54]. This brings into play the GPs’ need for specialist support. In our study, all the groups seemed to cooperate with PCTs at some level, and even the highly involved GPs relied on advice from PCTs. Accumulated evidence indicates that primary care needs such support from specialist PCTs to provide good quality palliative care [55, 56].

We found that the GPs displayed different levels of involvement in palliative care, and there was no consensus about their formal role. As early as in the 1990s, it was pointed out that the evolvement of PCTs allowed for the “blurring of roles” and that GPs felt that the patients were “taken over” by the PCTs [40, 57]. More recently, Wyatt et al. demonstrated unclarity of the GP’s role in end-of-life care and lack of a consensus of the GP’s role among the GPs themselves [56]. Our findings suggest that the GPs’ views about their own role is linked to how they perceive the role of the PCTs. Whereas the highly involved GPs described a close collaboration with the PCTs as an advisory resource, the less involved GPs described the PCTs as in charge of care, with the GP being on the side-line. This may suggest that it is not irrelevant how the collaboration between the GPs and the PCTs is undertaken. Evidence suggests expanding specialized palliative services is done at the expense of GPs’ ability to participate and maintain essential competencies in palliative care [56]. These findings pinpoint a central premise that is also evident in our study: GPs’ behaviour cannot be seen in isolation from the partners they collaborate with, as views about the GPs’ role rest in part on what they perceive to be expected of them. Thus, GPs’ general positive attitudes about palliative care do not by themselves determine the GPs’ degree of involvement, as these subjective normative beliefs must be taken into account [58].

Our findings indicate that GPs have skills and knowledge that are unique to them. However, previous findings indicate that GPs could be bypassed when the community nurses get direct access to the PCT physicians, perceiving them as more skilled and more available [54]. Such deficient practices do not only put the GP on the side-line [54], but also indicate that the value of the GPs’ contributions is not acknowledged. We argue that GPs’ skills and competencies seems to be complementary to those of PCT physicians, in much the same way as between GPs and municipal oncology nurses [54]. This merits a focus on including the GPs in the multidisciplinary approach to the palliative patients.

We found that the less involved GPs also experienced unclarities in the clinical handover of palliative patients from the hospital specialists. For palliative patients, care transitions represent a particular challenge, and the timely exchange of necessary information is vital [49].

The tendency for rural GPs to be more involved than urban GPs in palliative care must be interpreted with caution due to our sample size. This is, however, in line with our previous questionnaire study, showing that rural GPs to a larger degree reported to be central in palliative care [25]. In an Australian study, rural and remote GPs were found to have more responsibility for their palliative patients and less support from the PCTs than their urban colleagues [40]. Growing evidence thus suggests that geography plays a part in the division of tasks between PCTs and GPs, in turn possibly reinforcing these differences, resembling the “cycle of causation” described by Wyatt et al. [56]. Such a mechanism could possibly lead to the enabling of rural GPs, accessing the PCTs as a remote resource, and the deskilling of urban GPs, being put on the side-line of PCTs that provide more of the care in urban environments. In Norway, although conforming to national legislation, the practical organization of primary care in different municipalities varies, and steps to accommodate for a longer travel distance from the hospital may be appropriate. For the hospital specialists, however, our findings seem like a departure from the ideal of equality of services, strengthening our suggestion of unwarranted variation in the specialist service provision from the findings in our previous study [25]. These connections may need further investigation. Furthermore, it seems unnecessarily costly to let specialist services perform tasks that primary care demonstrably could manage, and this also challenges the principle of lowest effective level of care set by the Norwegian government [59]. To be able to meet the requirements, GPs do however need sufficient time and resources, which is not the case for many GPs in Norway today [60].

Salient in all the interviews was a relatively weaker, or total lack of, specialist support for palliative patients with non-cancer diagnoses, in particular for multimorbid patients. Our material thus demonstrates the perseverance of the view of palliative care in general, and PCTs in particular, as relevant mostly for cancer patients. This is in breach with the definitions of palliative care [61] and represents a problem for the timely provision of palliative care to all patients in need, irrespective of diagnosis [62].

## Conclusions

This study has shown that GPs encounter patients needing palliative care. They have evident strengths that could be important in the provision of palliative care for their patients. They rely on general medical knowledge and may need specialist support. The GPs we interviewed did not have a clear consensus about their role in palliative care. Multiple factors, including attitudes, collaboration, and clinical handover, seem to interact in complex ways to determine how GPs perceive their role and to what degree they are involved in palliative care.

Strengths, such as having a longitudinal, personal relationship with the patients and the continuity of care, may be unique to the GP, thus providing skills and knowledge complementary to the specialized skills of the PCT physician. Specialized teams with extensive outreach activities should be aware of the potential they have for both enabling and deskilling the GPs they collaborate with.

## Supplementary Information

**Additional file 1.** Interview guide focus group interviews; Palliative care in primary health care.

## Data Availability

The datasets generated and analyzed during the current study are not publicly available. Norwegian legislation requires data to be stored on a password-protected file on a university server for reasons of confidentiality and privacy. Access to the data is restricted to a period after completion of the project. Data could be available from the corresponding author on reasonable request.

## Abbreviations

ACP: Advance Care plan
CME: Continuing Medical Education program
EAPC: European Association for Palliative Care
GP: General Practitioner
IPA: Interpretative Phenomenological Analysis
OOH: Out-Of-Hours
PCT: Palliative Care Team
RGP: Regular General Practitioner
WHO: World Health Organization
