# Supplementary material for: Finding their place – general practitioners' experiences with palliative care—a Norwegian qualitative study
Source: BMC Palliat Care. 2022 Jul 12;21:126. doi: 10.1186/s12904-022-01015-1 (PMC9277777; doi:10.1186/s12904-022-01015-1)
Supplement: Supplementary file 1 — Additional file 1. Interview guide focus group interviews; Palliative care in primary health care. [file 12904_2022_1015_MOESM1_ESM.docx]

**Interview guide focus group interviews; Palliative care in primary health care**

Objective of the focus group study: Exploring how GPs define – and think about - their role and challenges in working with palliative care patients in primary care (PC). Their attitudes to the guideline is an area of interest. The study aims to elaborate on some aspects/ resultsfrom the previous questionnaire survey, as well as to gain new knowledge from the 1st person perspective.

**Part 1:**

**GPs' general experience with palliation**

Start by encouraging the GPs to come up with patient stories from their own practice. The participants are instructed to anonymize the patient/situation during the interview.

Can you think of /tell about patients where you as a GP have been involved in a palliative care trajectory? Suggested follow-up questions; Specifically request patient histories where the patient died at home. (link to the survey where this was rare)

**The role of GPs in palliative care**

How do you define and think about your GP's role in palliative care in primary care?

Suggested follow-up questions: Is a GP a key or peripheral player? (referring to survey where 50% of GPs say they are a key player)

What challenges have you faced in this work (or imagine, if little experience with palliative patients)? (keywords: competence/time/availability..)

Possible follow-up question: What is it realistic that the GP contributes to the current health service? What would be an ideal role/ contribution from the GP – if this could be facilitated?

What is the most important thing the GP can contribute to these patients?

(keywords/topics that may be requested if not come up by themselves in the discussion; availability, medication in palliative care, coordinating role, continuity, know the patient/family from before, support relatives, witness/ companion)

Can you refer to some significant insights – or learning situations in working with palliative care patients that were actually useful?

Suggested follow-up questions; What did you actually need help with – and where did you get that help? What did you learn that was actually useful?

**Part 2**

**GPs' experiences with and views on the guideline for palliative care**

Explore the GPs’ knowledge of – and thoughts around – a comprehensive guideline. The guideline is displayed in a hard copy — briefly refers to the scope/number of pages (190) and title.

How many of you have heard of this/know about this guideline?

What do you think about the scope of this guideline (given that there is a relatively small patient group but at the same time a complex subject area)?

Can we take a look together at the main content and discuss it together?

(Reads table of contents — or briefly refers to guideline highlights concerning GPs’ role)

What do you think about what it says here?

Suggested follow-up questions; discuss some specific topics in the policy; in particular, highlight the role of the GP and requirements for competence in the guideline.

Is there anything you miss – that hasn't been mentioned/discussed in the guideline?

Is the guideline the answer to GPs' challenges?

– and if not – what do GPs need to do the job?
